# Counseling Is a Relationship Not Just a Skill: Re-conceptualizing Health Behavior Change Communication by India’s Accredited Social Health Activists

**DOI:** 10.9745/GHSP-D-20-00426

**Published:** 2020-09-30

**Authors:** Rajani Ved, Kerry Scott

**Affiliations:** a National Health Systems Resource Centre, New Delhi, Delhi National Capital Region, India.; b Department of International Health, Johns Hopkins Bloomberg School of Public Health, Baltimore, MD, USA.

## Abstract

The capacity for India’s community health workers—accredited social health activists (ASHAs)—to promote healthy behaviors must be understood within the health system and community context. Their ability to influence health behaviors depends on the strength of their relationships with families and support they receive from the health system.

## Key Messages

- As both community members and health system functionaries, the capacity of India’s community health workers—accredited social health activists (ASHAs)—to improve health outcomes depends on their relationships with families and the support they receive from the health system.
- Training ASHAs on interpersonal communication, such as how to convey the right messages in a persuasive manner, is an important step to improve their impact as health behavior change communicators. However, it is not sufficient.
- Effective counseling also requires that ASHAs have strong positive relationships with community members. These relationships are developed over time as ASHAs link communities to high quality health services and showcase their capacity to directly meet key health needs.

See related article by Smittenaar et al.

Now in its fifteenth year, India’s accredited social health activist (ASHA) community health worker (CHW) program demonstrates all the opportunities and challenges that come with operating CHW programs at a massive scale, in this case with close to a million ASHAs. In this article, we discuss the tensions and benefits associated with ASHAs being both health system actors and community members and how policy and social relationships can support or undermine ASHA ability to improve community health.

The ASHA program is a part of a global re-emergence of national CHW programs,^1^^,^^2^ with the potential of CHWs to contribute to the Sustainable Development Goals around primary health care now well established in the academic literature^3^ and enshrined in World Health Organization guidelines.^4^

The article by Smittenaar et al.^5^ in this issue of GHSP is an important contribution to our understanding of CHW performance and impact for 2 reasons. First, this research examines a mature government CHW program operating at scale rather than of a small pilot or non-governmental organization initiative operating with higher resource inputs in non-generalizable settings. Second, this large-scale survey provides a uniquely rich data set that positions ASHAs within their community environment by assessing indicators from ASHAs, mothers, husbands, and mothers-in-law. The latter 2 categories have not been studied previously at scale in relation to ASHA reach and communication.

As health system actors, ASHAs are only as effective as the system that supports them. Individual-level factors related to ASHA performance, such as ASHA knowledge, must be contextualized within the health system support context, such as the training and continuing supervision they receive. ASHA time-use is closely tied to the health system context because ASHAs will decide whether to perform a role based on financial incentives, transportation considerations, competing demands, and also their self-assessment of their capacity to meet the family’s needs and expectations.

The article reported that receiving home-based newborn care was associated with positive health behaviors (clean cord care and exclusive breastfeeding). The study also found that only 31% of mothers were receiving adequate home visits in the first week, and ASHAs spent only 8.8% of their time on home visits to postpartum women. This care provision gap is striking, considering that home visits are 1 of the 5 key roles identified for ASHAs, alongside coordinating Village Health and Nut-rition Days, convening the Village Health and Sanitation Committees, accompanying patients to health facilities, and maintaining basic records. The 2013 ASHA guidelines include clear directions for when ASHAs should visit postpartum women. Financial incentives for providing home-based newborn care are quite substantial and are being claimed by a significant proportion of ASHAs; for example, in FY 2019–2020, data from the state’s reporting system show that about 76% of Uttar Pradesh’s ASHAs reported carrying out home-based newborn care visits. Further investigation is needed to understand the contradiction between this study’s finding of low coverage and the policy-level emphasis on ASHAs conducting home-based newborn care. Low coverage was also identified as a challenge in a study on postnatal home visits that analyzed large-scale surveys.^6^ The key questions to our mind relate to health systems functionality. Are ASHAs skipping home visits due to a lack of equipment or training that has undermined their motivation and makes them feel that they have little to offer during these visits? If an ASHA lacks a weighing scale, thermometer, and watch, or is not confident that she can adequately assess a newborn, she may feel there is no point in performing a home visit. To what extent does this contradiction call for bolstered accountability and oversight of ASHAs? Although the financial incentive that ASHAs receive for performing home-based newborn care is an important policy lever, it is clearly insufficient in ensuring that these visits occur.

As community members, ASHA relationships and interactions with families are subject to the broader power relations that shape social norms and behaviors. There is great value in studying CHWs as social actors engaged within power systems, as the Smittenaar et al. manuscript does. Just as ASHAs work within a health system context, they also work within community systems where they must navigate social relationships mediated by gender, age, caste, and other social hierarchies. Smittenaar et al. highlight some of the ways in which ASHAs navigate these relational dynamics, especially around targeting counseling to husbands and mothers-in-law. The broader community relationships and norms that ASHAs navigate require further exploration. Caste dynamics play out across almost all facets of rural life and may also influence ASHA acceptance into family homes or willingness to visit certain families. Community norms that prohibit outside visitors from interacting with newborns may be a major barrier facing ASHAs when trying to carry out home-based newborn care visits.

Many ASHAs have accrued strong social capital in their communities, and are seen as a vital link to the health system. An ASHA’s ability to maintain this social capital hinges in part on the health system’s performance, such as how families are treated by health facility staff during childbirth after they have been encouraged by the ASHA to have an institutional birth. ASHA efforts to maintain relationships and build social capital may be manifested in the large amount of time spent on accompanying women to health facilities. When the ASHA program began in 2005, ASHAs needed to physically attend a birth to receive their Janani Suraksha Yojana incentive payment. However, this policy changed some years later. ASHAs do not need to accompany women in labor; instead women just have to confirm that their ASHAs supported them in having an institutional delivery. And yet, as shown in the analysis by Smittenaar et al., the practice of accompanying women in labor persists and is associated with higher quality of care for women. Thus, this ASHA behavior endures beyond financial motivation and appears grounded in ASHA efforts to secure good care for women, maintain relationships, and meet community expectations.

As India moves toward primary health care reform, ASHAs are being mobilized to expand beyond maternal, child, and reproductive health into noncommunicable disease and mental health care. Although financial incentives for this additional work will be required, they must be coupled with the training on interpersonal communication called for by Smittenaar et al. But it is important that interpersonal communication be understood within the health system support and community context. Effective counseling is not just about messages or communication styles. It is not just a skill that can be taught. Instead, effective counseling occurs within a relationship of trust between the ASHA and beneficiary, which is built over time as ASHAs link up to functional health services and accrue social capital in the community. This is particularly true for entrenched normative behaviors, such as newborn care or diet or those related to stigmatized conditions, such as around mental health. ASHAs are best able to improve community health when they are skilled counselors who also earn community trust through directly providing some health services and supporting beneficiaries in accessing good quality higher-level care.
